# Optimizing Semantic Pointer Representations for Symbol-Like Processing in Spiking Neural Networks

**DOI:** 10.1371/journal.pone.0149928

**Published:** 2016-02-22

**Authors:** Jan Gosmann, Chris Eliasmith

**Affiliations:** Centre for Theoretical Neuroscience, University of Waterloo, Waterloo, Ontario, Canada; University of Sheffield, UNITED KINGDOM

## Abstract

The Semantic Pointer Architecture (SPA) is a proposal of specifying the computations and architectural elements needed to account for cognitive functions. By means of the Neural Engineering Framework (NEF) this proposal can be realized in a spiking neural network. However, in any such network each SPA transformation will accumulate noise. By increasing the accuracy of common SPA operations, the overall network performance can be increased considerably. As well, the representations in such networks present a trade-off between being able to represent all possible values and being only able to represent the most likely values, but with high accuracy. We derive a heuristic to find the near-optimal point in this trade-off. This allows us to improve the accuracy of common SPA operations by up to 25 times. Ultimately, it allows for a reduction of neuron number and a more efficient use of both traditional and neuromorphic hardware, which we demonstrate here.

## Introduction

The Neural Engineering Framework (NEF) [1] is a mathematical theory of how biological neural systems can implement a wide variety of dynamic functions. These methods have been used to propose novel models of a wider variety of neural systems, including the barn owl auditory system [2, 3], parts of the rodent navigation system [4], escape and swimming control in zebrafish [5], tactile working memory in monkeys [6], and simple decision making in humans [7] and rats [8]. In short, the NEF provides a method for capturing *how* neural computations might be performed. However, it does not specify what those computations are. More recently, the NEF has been used to underwrite a proposal regarding the mammalian neural architecture [9]. This proposal is called the Semantic Pointer Architecture (SPA), and suggests specific computations, architectural elements, and methods of representing and transmitting information to account for perceptual, motor, and cognitive behaviour. The SPA was used to construct what remains the largest functional brain model, called Spaun [10].

The SPA employs a small number of mathematical operations (discussed in detail later). When those operations are implemented in a neural network, each transformation will accumulate noise. Thus, any improvement to the accuracy of these operations will improve the overall network performance by a significant factor. The goal of this work is to improve the accuracy of such computations for a given amount of neural resources. We will show that such an improvement is possible by considering the distribution of values represented in neural ensembles. By allowing a less accurate representation of rarely occurring values we can considerably improve the representation of common values. For example, if almost all represented values had a magnitude below one, it is not useful to have neurons tuned to represent values with a larger magnitude. In fact, we derive a heuristic to determine a near optimal trade-off of being able to represent all possibly occurring values and only being able to represent the most likely values. Identifying this heuristic allows for much more efficient simulation of neural components that perform typical SPA operations because less neurons can be used while maintaining the error level. Large-scale models require optimizations of this sort because they are computationally costly to simulate. For instance, the Spaun model required 2.5h of simulation time for each second of simulated time. With fewer neurons, model simulation can run faster or larger models can be run without increased hardware requirements. As well, such optimizations make it more feasible to put computationally useful networks on non-traditional, neuromorphic hardware.

While traditional von Neumann computers approach physical limits, neuromorphic platforms promise continuing speed-ups and a far better energy efficiency. Projects like CAVIAR [11], SyNAPSE/TrueNorth [12], and Neurogrid [13] to scale up these platforms are underway. Nevertheless, more effective usage of the hardware reduces costs and allows to run larger or more complicated models on the same hardware. While it is common to analyze the complexity of algorithms on traditional platforms to optimize their efficiency, it is not very common on neuromorphic hardware, presumably because many neural network approaches do not allow for analytic investigation of the computations.

Choudhary et al. [14], Mundy et al. [15], and Wang et al. [16] have shown that the Neural Engineering Framework (NEF) [1] is a viable method to translate the mathematical formulation of an algorithm into a neural network which can run on neuromorphic hardware. Consequently, optimizations of the sort we perform here will benefit this growing body of work on neuromorphic computation by providing methods for a more efficient usage of the available hardware resources.

The paper is organized as follows: First, we give an introduction to the Neural Engineering Framework and the Semantic Pointer Architecture before describing our optimization methods in detail. Following that, we test the methods first with computer simulations of standard leaky integrate-and-fire neurons and then with a SpiNNaker neuromorphic implementation using fixed-point calculations. To show the applicability to large scale models, we demonstrate the improvement on a recent model of the *n*-back task. Finally, we discuss the results, noting that these methods can reduce the number of neurons used by up to 97.5%.

## Methods

### The Neural Engineering Framework (NEF)

The methods in this paper are applicable to the Neural Engineering Framework (NEF) [1] which allows the construction of large-scale neural networks from a mathematical description. The NEF is based on three core principles:

1. Representation: Populations of neurons represent vectors by non-linear encoding and linear decoding.
2. Transformation: Functions of time-varying variables can be computed by an alternative linear decoding of the vector represented by a neural population.
3. Dynamics: Represented values can be treated as state variables (see Principle 1). A dynamical system of these state variables can be implemented with recurrent connections. Necessary nonlinearities can be computed with principle 2.

**Fig 1:**
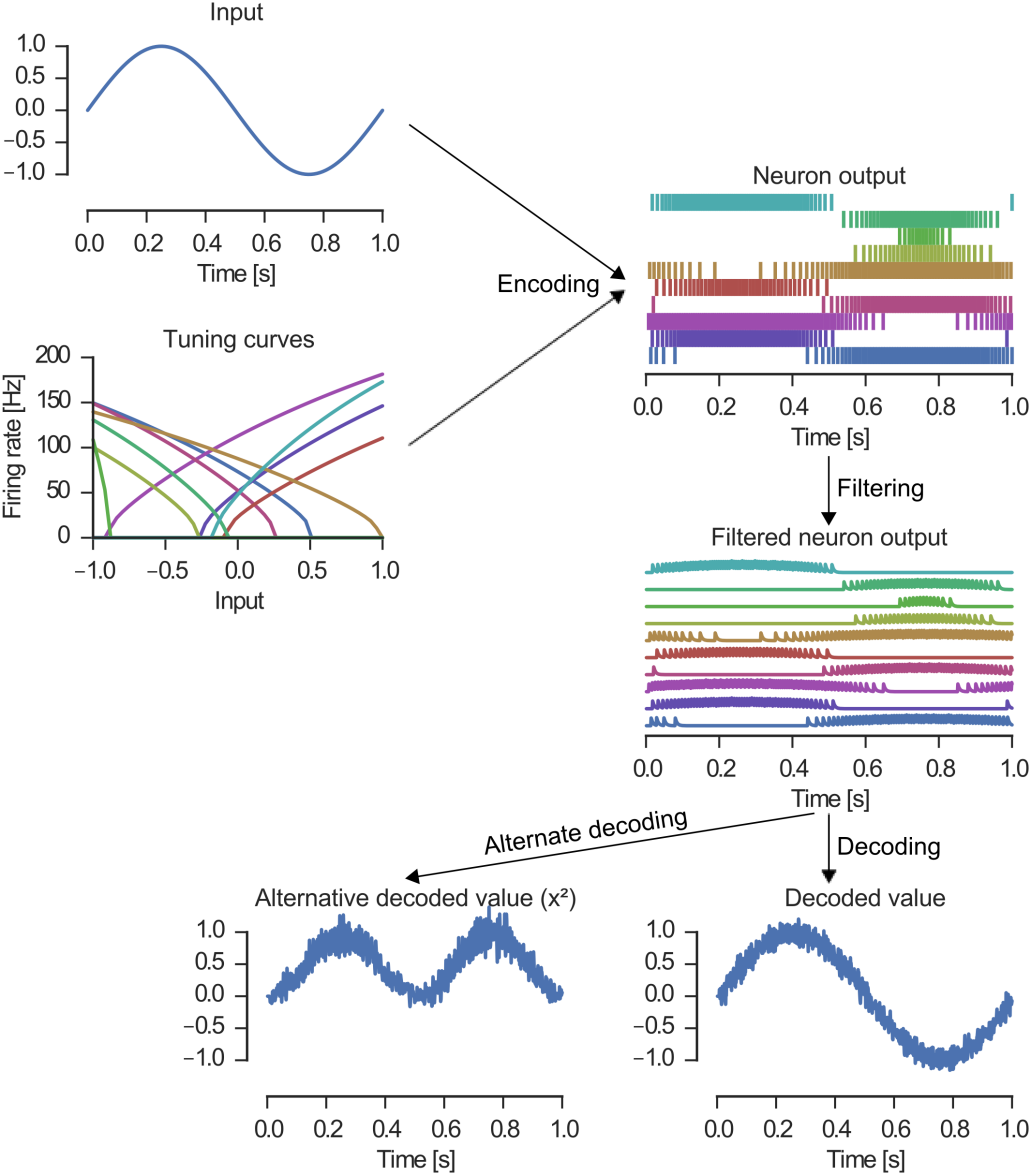
Representation and transformation in the Neural Engineering Framework (NEF). The NEF specifies how an input (upper left) is encoded by a population of neurons with individual tuning curves. The orientation of the rising flank of the tuning curve corresponds to the preferred direction ***e***_*i*_ of the neuron (either −1 or +1 in this one-dimensional example). The encoding equation will typically produce spike trains for the neurons (top right). To decode the represented value, the spike trains are filtered (corresponding to synaptic filtering) and then a linear weighted sum is computed (bottom right). Using different sets of decoding weights non-linear transformations can be implemented (bottom left).

Here we focus on the first two principles. NEF models typically begin by suggesting a description of the cognitive system using time-varying, real-valued vectors and transformations of these vectors. The NEF specifies a population encoding given a group of neurons for such a vector. It also specifies how to approximately decode the vector or a transformation of it from the population coding of such a group of neurons. By combining encoding and decoding, populations of neurons can be connected to create networks to transmit and process information. In the following we will detail these steps (see also Fig 1).

The representation of a time-varying vector ***x***(*t*) by a neuron *i* is given by the non-linear encoding

$$\begin{array}{c} a_{i}\left(t\right)=G\left[J\left(x\left(t\right)\right)\right],\;J\left(x\left(t\right)\right)=\alpha_{i}\left(e_{i}\cdot x\left(t\right)\right)+J_{\text{bias},i} \end{array}$$ (1)

where *a*_*i*_(*t*) is the neuron’s activity, *G*[⋅] a non-linear activation function, *J*(***x***) the incoming current to the neuron, *α*_*i*_ a gain factor, ***e***_*i*_ the neuron’s encoder or preferred direction vector, and *J*_*bias*, *i*_ the background current to the neuron. The dot product ***e***_*i*_ ⋅ ***x*** with the neuron’s encoder accounts for the fact that a neuron is typically tuned to specific stimuli.

Depending on the activation function, the activity *a*_*i*_(*t*) can be a rate approximation or a spike train ∑_*k*_ δ(*t* − *t*_*k*_) expressed as a sum of Dirac delta δ functions for all spikes *k* at times *t*_*k*_. A specific neuromorphic hardware platform may dictate *G*[⋅] or place constraints on it. If the activation function returns a spike train, a continuous signal can be obtained by convolving the spike train with a filter that accounts for post-synaptic effects in a receiving neuron. In this work we use an exponential decay of the form exp(−*t*/*τ*), where *τ* is the post-synaptic time constant for a given neurotransmitter.

For simplicity, we assume *a*_*i*_ to be a rate approximation in the following equations. The value represented by a group of *N* neurons, called *ensemble* in the NEF terminology, can be estimated by a weighted linear decoding of their activities *a*_*i*_ as

$$\begin{array}{c} {\hat{x}}_{k}\left(t\right)=\sum_{i=1}^{N}d_{ik}a_{i}\left(t\right) \end{array}$$ (2)

with decoding weights *d*_*ik*_. Together, the encoding and decoding equations specify the first NEF core principle of representation.

The decoding weights are typically found by a least-squares optimization of the difference $\|\hat{x}-x\|$ with *Q* evaluation points ***x*** randomly picked from the desired representational range. This range is usually chosen to be a hyper-sphere with radius *r*. Thus, all vectors with a length of less than *r* will be represented equally well on average, but vectors with a length exceeding *r* will have an increasing error in their representation as the decoding weights have not been optimized for this range. Unfortunately, arbitrarily increasing the radius will spread out the constant number of evaluation points over the representational range which will lead to a less precise decoding for vectors within range. Because of this, the choice of *r* can be quite important and the methods presented here are concerned with finding a good radius *r* considering the statistical distribution of represented values.

To solve the least-square problem for the decoding weights, we define the matrices

$$\begin{array}{c} A=[\begin{array}{cccc} a_{1}\left(x_{1}\right) & a_{1}\left(x_{2}\right) & \cdots & a_{1}\left(x_{Q}\right)\\ a_{2}\left(x_{1}\right) & a_{2}\left(x_{2}\right) & \cdots & a_{2}\left(x_{Q}\right)\\ \vdots & \vdots & \ddots & \vdots \\ a_{N}\left(x_{1}\right) & a_{N}\left(x_{2}\right) & \cdots & a_{N}\left(x_{Q}\right) \end{array}]\;\text{and}\;X=[\begin{array}{c} x_{1}\\ x_{2}\\ \vdots \\ x_{Q} \end{array}] \end{array}$$ (3)

and use the regularized pseudo-inverse as follows

$$\begin{array}{c} \left[\begin{array}{c} d_{1}^{⊤}\\ d_{2}^{⊤}\\ \vdots \\ d_{N}^{⊤} \end{array}\right]={\left(AA^{⊤}+Q\gamma^{2}\text{max​}{\left(A\;\right)}^{\;2}I\right)}^{-1}AX \end{array}$$ (4)

where max(***A***) is the largest element in ***A***. The scale of regularization is denoted by *γ* and **I** represents the identity matrix.

Next, we consider the principle of transformation. The decoding weights for an arbitrary function *f*(*x*) can be computed by changing ***X*** in Eq 3 to

$$\begin{array}{c} X=[\begin{array}{c} f(x_{1})\\ f(x_{2})\\ \vdots \\ f(x_{Q}) \end{array}]. \end{array}$$ (5)

This produces a different set of decoding weights transforming the represented value. Note that even though the decoding is linear, it can approximate non-linear functions.

Individual ensembles can be connected to form larger functional networks in the NEF. To do so we determine the decoding weights for the desired transformation from the presynaptic ensemble. The decoded value can then be fed to and encoded in the postsynaptic ensemble. Mathematically this gives an outer product of the presynaptic decoding weights and postsynaptic encoders which specifies the synaptic connection weights from *j* to *i* as

$$\begin{array}{c} W_{ij}=e_{i}^{⊤}Pd_{j} \end{array}$$ (6)

where the matrix ***P*** can give a linear transformation of the represented vector to be implemented in the connection weights. To implement a communication channel the identity matrix is used.

Because the third principle of dynamics is not relevant to the methods in this paper, we skip a detailed description for the sake of brevity and refer the interested reader to [1].

### The Semantic Pointer Architecture (SPA)

Many cognitive models implemented with the NEF, including Spaun, use the Semantic Pointer Architecture (SPA) [9]. The SPA consists of a variety of components, computations, and representational strategies thought to reflect neural processing in the mammalian brain. In general, the representations used throughout the SPA are called *semantic pointers*. One aspect of the SPA that is important for cognitive processing is the representation of structured information in spiking neurons. The semantic pointers used in the SPA for cognitive processing are based on Holographic Reduced Representations (HRR) proposed by Tony Plate [17], which is one of a family of representational schemes collectively referred to as Vector Symbolic Architectures (VSA) [18]. These semantic pointers are usually normalized to unit length. This normalization constraint is a basic assumption of the following optimization methods. Multiple semantic pointers can be combined with addition to obtain a new semantic pointer similar to each individual item. As with HRRs, the SPA also defines a binding operation that employs circular convolution:

$$\begin{array}{c} u=v\;⊛\;w:\;u_{i}=\sum_{j=1}^{D}v_{j}w_{(i-j)\;\mathrm{mod}\;D}. \end{array}$$ (7)

The binding produces a new vector dissimilar to the original vectors. From the resulting vector, the operands can be approximately recovered by a circular convolution with the involution of the other operand. That is,

$$\begin{array}{c} v\approx u\;⊛\;w^{-1} \end{array}$$ (8)

with the involution defined as

$$\begin{array}{c} w^{-1}=\left(w_{1},w_{D},w_{D-1},\cdots ,w_{2}\right). \end{array}$$ (9)

Furthermore, a vector ***v*** with |***w***| = |***v*** ⊛ ***w***| is called unitary. Note that the circular convolution is an element-wise multiplication in the Fourier space, which allows for a well-characterized implementation in neurons with the NEF. The transformation to and from Fourier space is linear and can thus be implemented in the connection weights between neural ensembles (see Eq 6). The multiplications are non-linear, but can nevertheless be implemented accurately with the NEF [19].

Using addition and circular convolution, multiple semantic pointers can be stored and retrieved within a single vector. Given semantic pointers for SQUARE, CIRCLE, BLUE, and RED, a scene with a blue square and a red circle could be represented as

$$\begin{array}{c} scene=\text{SQUARE }⊛\text{ BLUE}+\text{CIRCLE}⊛\text{RED}. \end{array}$$ (10)

From this the color of the square can be obtained with the involution as

$$\begin{array}{c} {\text{SQUARE}}^{-1}\;⊛\;scene\approx \text{BLUE}+\text{noise}. \end{array}$$ (11)

Finally, semantic pointers ***v*** and ***w*** can be compared using the dot product ***v*** ⋅ ***w*** which will give a measure of similarity that lies between -1 to 1.

This characterization of structured representation in the SPA has been used to build a variety of spiking neural models that simulate simple linguistic parsing [20], the Wason card task [21], and human performance on the Raven’s Progressive Matrices [22], a general intelligence test. In each case, a large proportion of the computational resources are dedicated to computing the binding operation, which is essentially a high-dimensional product. This work focusses on improving the accuracy of such computations for a given amount of neural resources.

### High-dimensional representations

To calculate the optimal decoding weights in the NEF it is necessary to invert an *N* × *N* matrix. The required time for this inversion grows cubic with *N*. As long as no non-linear combination of the represented vector components has to be calculated it is possible to split the vector into *s* subvectors and represent each subvector with an individual neural population. When keeping the number of neurons fixed, this requires only *s* inversions of *N*/*s* × *N*/*s* matrices with a complexity of *O*(*N*^3^/*s*^2^) instead of *O*(*N*^3^). Note that in most models based on the NEF and SPA linear combinations of vectors components, which allow this optimization are extremely common. Moreover, *N* and *s* are usually chosen in dependence of the total number of dimensions *D*. In that case the improvement is not just a constant factor, but changes the asymptotic behaviour (e.g. with *N* = *kD* and *s* = *D* we would have *O*(*D*^3^) vs. *O*(*D*)).

Furthermore, if we increase the number of dimensions *D*, we usually want to keep the total error caused by neuron noise constant. If the complete *D*-dimensional vector is stored in one ensemble, the number of neurons *N* has to be scaled by *D*^2^ to achieve this goal. By splitting up the vector into *s* subvectors with a fixed number of components (e.g. one and thus *s* = *D*), the number of neurons only needs to be scaled linearly with the number of dimensions. (See S4 Appendix.)

In the NEF, each ensemble is usually optimized over a certain radius *r* by randomly picking evaluation points from a hyper-sphere. In case of cognitive semantic pointers this radius is typically unit length. However, when splitting up semantic pointers in subvectors, those subvectors will not be of unit length anymore. This results in ensembles being optimized over irrelevant areas of the input space (all vectors up to unit length) and decreases the accuracy in the relevant range (only the vectors with length of the subvectors). Moreover, some NEF-type models rely on a normalization behavior outside of the optimization radius (e.g. [10]). By splitting up the vector, but keeping the radius fixed, the normalization will no longer normalize to unit vectors, but considerably larger vectors.

In the following we present a method to optimize the ensemble radius in these cases to increase the network accuracy.

### Finding the optimal radius

The radius of the hypersphere from which evaluation points are chosen is central to the optimization proposed in this paper. If we chose the radius too small, values might fall outside of this hypersphere and they cannot be represented well because the neural ensemble has not been optimized for values in this range. If, however, we chose the radius too large some proportion the evaluation points will be used to cover an irrelevant part of the input space, i.e., a part where no values have to be represented. We want to find the radius with the best trade-off of these two effects.

We proceed by defining an approximate error function in dependence of the radius. By minimizing this error function, a nearly optimal radius can be obtained. There are three factors contributing to the representation error:

- Distortion *E*_*x* > *r*_ from points that fall outside of the optimization radius *r*.
- Distortion *E*_*x* ≤ *r*_ from points that fall inside of the optimization radius *r*.
- Noise from the spiking and random fluctuations of the neurons.

In the following we derive expressions for the static distortion given by the first two error contributions listed above. The neuron noise is assumed to be independent of the radius *r* and will be excluded in the analysis.

#### Distortion outside of the radius

Let ***v*** = (*v*_1_, …, *v*_*D*_) be a random vector with *D* = *n* + *m* independent components distributed according to $v_{i}∼N\left(0,\sigma^{2}\right)$. The probability density function (PDF) of the length of this vector is given by (see S1 Appendix)

$$\begin{array}{c} p_{\left|{}_{v}\right|}\left(x;\sigma ,D\right)=k_{D}x^{D-1}\text{exp​}\left(-\frac{x^{2}}{2\sigma^{2}}\right) \end{array}$$ (12)

with normalizing constant

$$\begin{array}{c} k_{D}=\frac{1}{2^{(D/2)-1}\sigma^{D}\Gamma \left(\frac{D}{2}\right)} \end{array}$$ (13)

where $\Gamma \left(t\right)=\int_{0}^{\infty }x^{t-1}\exp\;(-x)$ d*x* is the gamma function. This reduces to a half-normal distribution for *D* = 1 and a Rayleigh distribution for *D* = 2.

This distribution allows us to derive the distribution of the length $|{\hat{v}}_{1:m}|$ of a subvector with the first *m* components of a unit vector. To do so, one has to determine the quotient of the probability distribution corresponding to the following equation of random variables:

$$\begin{array}{c} \left|{\hat{v}}_{1:m}\right|=\frac{\left|v_{1:m}\right|}{\left|v\right|} \end{array}$$ (14)

**Fig 2:**
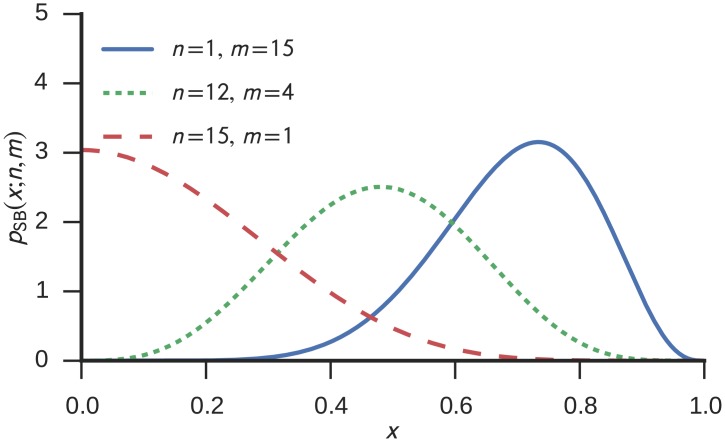
Examples of the SqrtBeta probability density function for different parameterizations.

The resulting probability distribution is given by

$$\begin{array}{c} p_{\text{SB}}\left(x;n,m\right)=\frac{2}{\text{B​}\left(\frac{n}{2},\frac{m}{2}\right)}{\left(x^{2}\right)}^{\left(m-1\right)/2}{\left(1-x^{2}\right)}^{n/2-1} \end{array}$$ (15)

with the beta function $\text{B​}\left(a,b\right)=\overset{1}{\underset{0}{\int }}t^{a-1}{\left(1-t\right)}^{b-1}dt$. We refer to this distribution as the *square root beta distribution* and use the subscript *SB* because of its close relationship to the beta distribution. In fact, the probability distribution of ${|{\hat{v}}_{1:m}|}^{2}$ is given by a beta distribution with parameters *α* = *m*/2 and *β* = *n*/2. Example plots of different parameterizations of the square root beta probability density function are shown in Fig 2.

The cumulative distribution function is given by (see S2 Appendix)

$$\begin{array}{c} \begin{array}{c} F_{\text{SB}}\left(x;n,m\right)=\frac{\text{B​}\left(x^{2};\frac{m}{2},\frac{n}{2}\right)}{\text{B}\left(\text{​}\frac{m}{2},\frac{n}{2}\right)} \end{array} \end{array}$$ (16)

with the incomplete beta function $\text{B​}\left(x;a,b\right)=\overset{}{\underset{}{\int_{0}^{x}t^{a-1}{\left(1-t\right)}^{b-1}\text{d}t}}$.

Assuming that every point outside of the optimization radius *r* gets projected onto the hyper-sphere surface with radius *r*, the squared error of a point ***y*** with *y* = |***y***| is (*y*−*r*)^2^. (Most neuron models used with the NEF will not do a hard cut-off at the radius, but saturate more slowly. Thus, values outside of the radius *r* can still be represented to a certain degree.) Weighting this expression by the probability density of *y* and renormalizing gives the error expression

$$\begin{array}{c} E_{x>r}=\frac{\int_{r}^{1}{\left(y-r\right)}^{2}p_{\text{SB}}\left(y;D-m,m\right)\text{d}y\;}{1-F_{\text{SB}}\left(x;D-m,m\right)}. \end{array}$$ (17)

The integral can be written with a number of beta functions (see S3 Appendix) which allows an easy implementation in software, as many libraries (e.g. SciPy) provide implementations of this function.

#### Distortion inside of the radius

The exact distortion inside of the radius is given by

$$\begin{array}{c} \begin{array}{c} \begin{array}{c} E_{x<r}^{*}=\frac{1}{\left|X\right|}\int_{X}{\left\|x-\overset{N}{\underset{i=1}{\Sigma }}a_{i}\left(x\right)d_{i}\right\|}^{2}\text{d}x \end{array}. \end{array} \end{array}$$ (18)

and can be estimated from our finite set of evaluation points as

$$E_{x<r}=\frac{1}{Q}\overset{Q}{\underset{q=1}{\Sigma }}\|{ry_{q}-\overset{N}{\underset{i=1}{\Sigma }}a_{i}\left(ry_{q}\right)d_{i}\|}^{2}.$$ (19)

where *y*_*q*_ are evaluation points sampled from the unit hyper-sphere *S*^*D*^.

#### Complete error function

Weighting both error contributions by the probability of representing a value in the respective domains gives the complete error function:

$$\begin{array}{c} E\left(r\right)=E_{x\leq r}F_{\text{SB}}\left(r;D-m,m\right)+E_{x>r}\left(1-F_{\text{SB}}\left(r;D-m,m\right)\right). \end{array}$$ (20)

**Fig 3:**
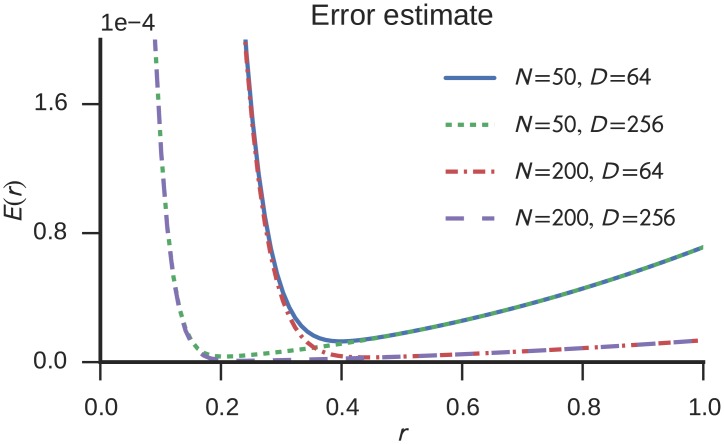
Estimated distortion error for different neuron counts *N* and vector dimensionality *D* in dependence of the ensemble radius *r*. It is assumed that each vector component is stored in an individual ensemble (*m* = 1).

See Fig 3 for a number of example plots showing how Eq 20 can be used to estimate the expected error for various numbers of neurons (*N*) and dimensions (*D*). The error function is well-behaved for numerical optimization methods as it is monotonically decreasing towards the minimum from either side of it. Thus, the optimal radius *r* can be found easily and efficiently.

## Results

To validate the derived error function we performed a number of simulations using the Nengo neural simulator [23]. For the simulations we generated a slowly varying *D*-dimensional semantic pointer by generating low-pass filtered white noise for each vector component and normalizing the resulting vector to unit length. The cutoff frequency was set to 5Hz. The simulation and data analysis code is available at https://github.com/ctn-archive/spaopt.

### Empirical distortion error

To empirically determine the distortion we used the rate approximation of leaky integrate-and-fire (LIF) neurons given by

$$G_{i}\text{​}\left[J_{i}\left(x\right)\right]=\{\begin{array}{ll} \frac{1}{\tau_{ref}-\tau_{RC}\text{ ln​}\left(1-\frac{J_{i}^{\text{thr}}}{J_{i}\left(x\right)}\right)} & J_{i}\left(x\right)>J_{i}^{\text{thr}}\\ 0 & \text{otherwise} \end{array}$$ (21)

with refractory time constant *τ*_ref_ = 2 ms, membrane time constant *τ*_*RC*_ = 20 ms, and threshold current $J_{i}^{\text{thr}}$ randomly chosen for each neuron to produce firing rates in the range of 200Hz to 400Hz. The use of rate neurons eliminates any spiking noise so that the difference of the decoded vector and the input vector is the actual distortion after correcting for delay in the synaptic transmission. A single ensemble representing *m* subdimensions of the *D* dimensional vector was simulated. After an initial 0.5s the error in every 1 ms time step of 20 trials with a duration of 10s each was averaged.

**Fig 4:**
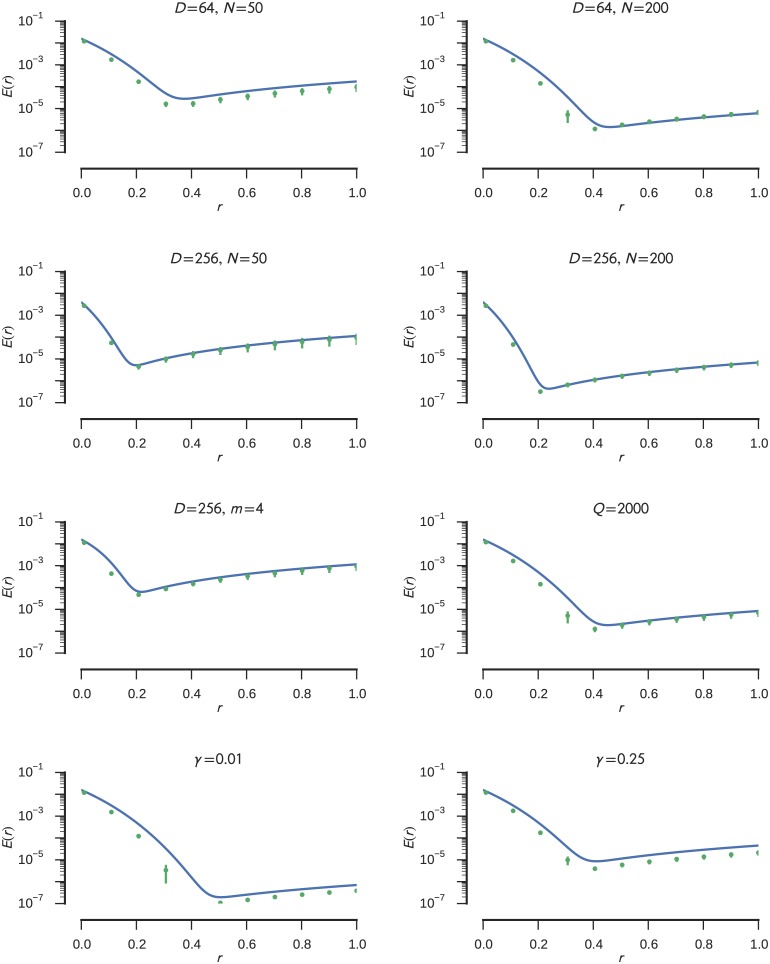
Comparison of the estimated distortion error (solid blue line) and empirically measured distortion error (green scatter points). Error bars on the scatter points denote the 95% confidence intervals. The empirical error is the mean of 20 trials with a duration of 10s each. See the text for details on how the empirical error was obtained. As long as not otherwise noted in the title of the individual plot the simulations were performed with *N* = 200 neurons, a vector dimensionality of *D* = 64, *m* = 1 subdimensions per individual ensemble, *Q* = max{2*Nm*, min{max{500*m*, 750}, 2500}} evaluation points, and a regularization of *γ* = 0.1.

The results for different parameter sets together with the analytical error estimate are plotted in Fig 4. The empirical error is closely matched by the analytical estimation for most tested parameter sets. Where this is not the case, especially for smaller radii, it tends to overestimate the error. This is most likely due to the assumption that all values outside of the radius get projected onto the radius. The actual neuron model saturates a bit slower which reduces the error. For large radii some deviation is introduced because the error estimate is based on a limited number of evaluation points which cannot cover the complete continuous input space. Consequently, the error function shown in Eq 20 is reliable, with a mean deviation of (0.248 ± 0.533) × 10^−3^ of the empirical error.

### Representation

**Fig 5:**
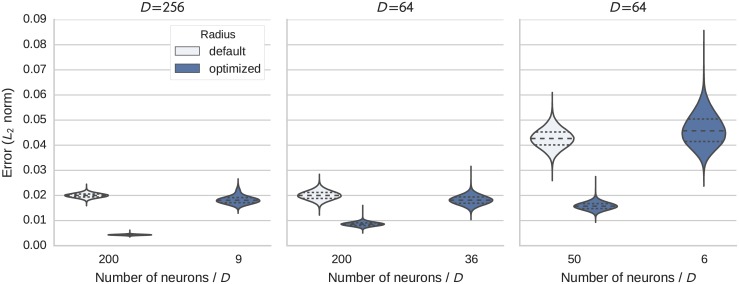
Distribution of the error (Euclidean distance) in the representation of a *D* dimensional vector. Each subplot shows the results for a fixed dimensionality. In each subplot results with the default and optimized radius for a baseline number of neurons per dimension are given and also the result for the optimized radius and heuristically reduced neuron number (see text for details).

Next we tested the accuracy of representation with spiking LIF neurons using the same neuronal parameters as for the rate neurons (*τ*_ref_ = 2 ms, *τ*_*RC*_ = 20 ms, random voltage thresholds $V_{i}^{\text{thr}}$ to yield firing rates in the range from 200Hz to 400Hz). A network of *D* ensembles each representing a single dimension was used. The root mean square error (RMSE) of the decoded vector was recorded in each timestep across 20 independent simulations. Fig 5 shows the distribution of the recorded error values. We use violin plots here which are similar to box plots, but additionally show the probability density providing additional information about the mean and variance. The horizontal lines mark quartiles.

In the test cases, the RMSE was reduced by a factor of 2.3 to 4.6 compared to using a non-optimized radius. As the mean square error is proportional to 1/*N* (Fig. 2.6 in [1]) this should allow us to reduce the number of neurons with the optimized radius by the square of these factors without an increase in the error compared to the default radius. We refer to this reduced number of neurons as the *heuristically reduced neuron number*, as it is our attempt to choose fewer neurons (per dimension) while retaining a similar level of error. Corresponding simulation results are included in Fig 5. Despite the reduction in the amount of neurons between about 5 to 22 times, the majority of the error distribution for the heuristically reduced neuron number is below the baseline distribution in most cases. Only for 64 dimensions with 50 neurons reduced to 6 neurons does the error distribution get slightly wider and exhibit a tail extending to larger error values. Presumably this is caused by other distortion effects with very few neurons not approximated by the simple 1/*N* rule.

### Circular convolution

To show that the radius optimization not only improves representational accuracy, but also the accuracy of transformations, we tested it with a circular convolution network. The same random and slowly varying input vector (normalized random vector with white noise components) was used as one operand and the second operand was fixed to a random unitary vector. Otherwise, the same procedure is used as in the representation test.

The circular convolution in Nengo is computed by taking the discrete Fourier transform (DFT), multiplying the Fourier coefficients in individual ensembles, and calculating the inverse discrete Fourier transform (IDFT). This characterization of the computation in the state space results in a simple, 2-layer feedforward network. The Nengo default implementation uses a normalization factor of 1 for the DFT, a factor of 1/*D* for the IDFT, and a radius of 2 for the multiplication ensembles. In the optimized implementation we use a normalization factor of $1/\sqrt{D}$ for both the DFT and IDFT to keep the coefficient vector at unit length and apply the radius optimization.

**Fig 6:**
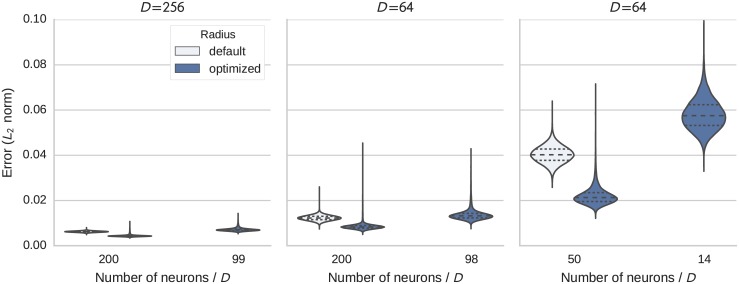
Distribution of the error (Euclidean distance) in the calculation of circular convolution with *D* dimensional vectors. Each subplot shows the results for a fixed dimensionality. In each subplot results with the default and optimized radius for a baseline number of neurons per dimension are given and also the result for the optimized radius and heuristically reduced neuron number (see text for details).

The resulting distributions of the RMSE with respect to the analytical circular convolution are shown in Fig 6. The optimized radius shifts the majority of the error distribution downward, but leaves a long tail. The majority of all occurring values can still be represented with the smaller radius. But there are a few rare instances where a vector has a few components larger than the average. These cannot be accurately represented with the smaller radius. Thus, by optimizing the radius we are able to get a better representation of most values at the cost of a worse representation for a few values.

In the test cases the RMSE was reduced by a factor ranging from 1.4 to 1.8. We applied the same heuristic as in the representation test to reduce the neuron number and included the results in Fig 6. The results are similar to the representation test. Given enough neurons to start with the reduction with the radius optimization gives an error distribution close to the baseline. Only when the number of neurons gets too low (e.g. from 50 to 14) does the heuristic becomes inaccurate resulting in an increase in error.

### Dot product

**Fig 7:**
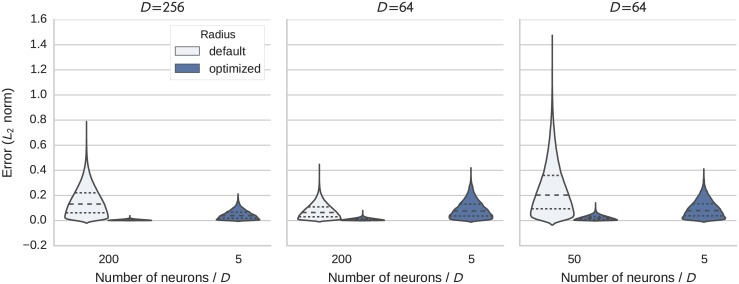
Distribution of the error (Euclidean distance) in the calculation of dot products with *D* dimensional vectors. Each subplot shows the results for a fixed dimensionality. In each subplot results with the default and optimized radius for a baseline number of neurons per dimension are given and also the result for the optimized radius and heuristically reduced neuron number (see text for details).

In addition to the circular convolution for binding, the SPA uses dot products for comparison of semantic pointers. In Nengo a dot product is implemented by multiplying the vector components in individual parabolic multiplier networks and having the outputs project to a single ensemble to generate a sum. The default ensemble radius is 1. Fig 7 shows the comparison of the default implementation and the optimized radius. Using the optimized radius shrinks the distribution’s variance and reduces the RMSE by a factor of 6.6 to 25.7 for the test cases. Here we also note a significantly different shape of the distribution because the dot product sums over all dimensions.

Again this optimization allows for the use of far fewer neurons with the optimized radius. We set a lower limit of five neurons per dimension as fewer neurons could easily give rise to additional error sources. The results of reducing the number of neurons by 10 to 20 times to this lower limit is depicted in the same figure. The error of the optimized dot product is still clearly below that of the default implementation.

### Neuromorphic hardware

As discussed in the introduction, these optimizations are helpful for improving the accuracy of computations being performed in resource-limited neuromorphic hardware. Here we verify that these optimizations hold on physical neuromorphic hardware by using the SpiNNaker platform [24]. The LIF neuron on this platform uses fixed point instead of floating point calculations as was assumbed by the previous simulations. The maximum number of dimensions in these tests was limited to 25 due to current limitations in the SpiNNaker implementation. A single SpiNNaker core is not able to generate all required packages to transmit within the required amount of time. These limitations are expected to be removed shortly by using multiple cores for the packet generation if needed.

**Fig 8:**
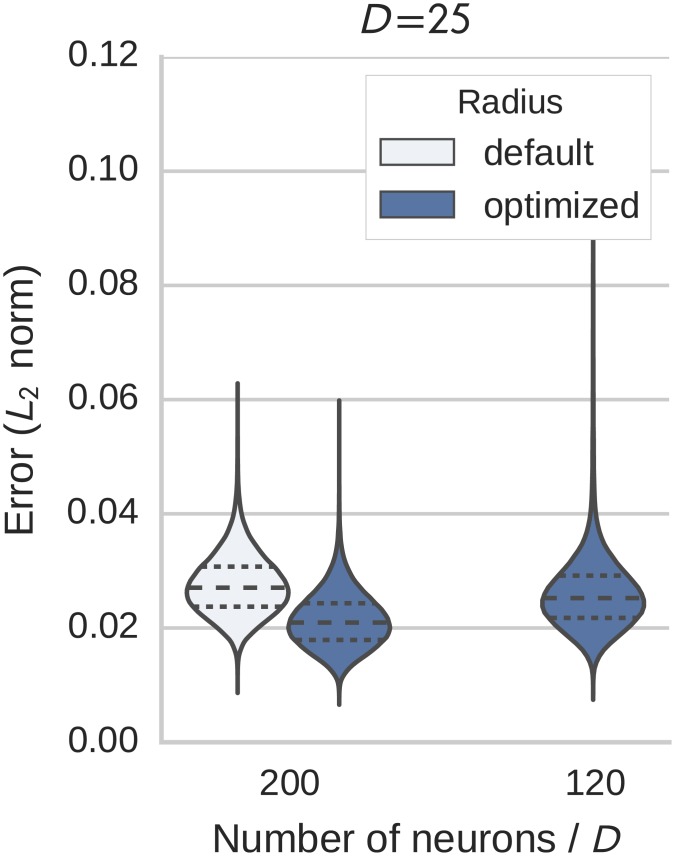
Distribution of the error (Euclidean distance) in the representation of a 25 dimensional vector on the SpiNNaker neuromorphic hardware platform. The results with the default and optimized radius for a baseline of 200 neurons per dimension are given and also the result for the optimized radius and heuristically reduced neuron number (see text for details).

The results in Fig 8 show a decrease of the RMSE by a factor of 1.3 with the radius optimization when representing a value (without transformation). This allows to decrease the number of neurons per dimension from 200 to 120 per dimension while still achieving an error comparable to the baseline. The tail of the distribution gets longer, but the total contribution to the mass of the distribution is negligible. Overall, this means that a network of 5000 neurons was reduced to 3000 neurons, while retaining the same performance.

### Large scale model

We have shown the effectiveness of the optimization method for single ensembles. To verify the improvements on larger scale models, we employ the optimization method on an NEF model of the *n*-back task, a test of executive control of working memory that achieves human-like performance [25]. In particular, the network makes use of dot products, circular convolutions, and recurrent memory populations. The model uses 92250 neurons overall, with 60600 representing vectors that allow for the proposed optimization method. The vector dimensionality was set to 64, and 3200 neurons were allocated to represent a single vector, except in dot products and circular convolutions where 6400 and 12800 neurons were used respectively. From input to output information passes through 11 neural populations with the optimization methods applied. This includes two circular convolutions and a dot product. Furthermore, the model includes recurrent processing among these 11 neural populations and a semantic pointer gets convolved *n* times (e.g., 2 times in the 2-back task) before it is read out.

**Fig 9:**
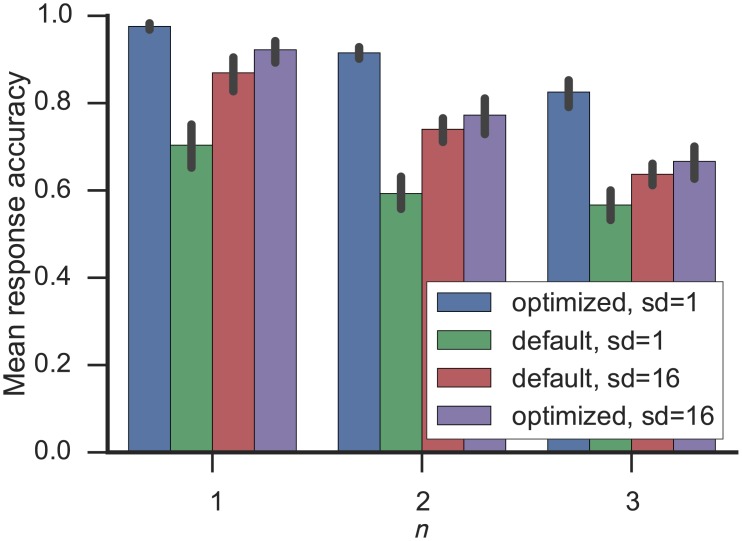
Model performance on the *n*-back task. Results for *n* ∈ {1, 2, 3} are shown. For each *n* results with (optimized) and without optimization (default) of the radius are presented. Each of these two conditions was simulated with using a single ensemble for each dimensions (sd = 1) and using a single ensemble for sets of 16 dimensions (sd = 16).

The original results were obtained with the optimization methods presented in this paper with a single ensemble for each vector component. If we disable the optimization of the radius and use the Nengo default, the effect on the model performance is detrimental (see Fig 9). However the default radius was chosen for a representation of 16 vector components in a single ensemble. Splitting up the representation of the vector in this way also yields a performance below the baseline. As a final comparison, using the radius optimization on this representation of 16 vector components each slightly improves the performance, but due to the low vector dimensionality of 64, which is only split into four parts, the improvement is not as large as when each vector component is represented individually. Based on the previous results on the improvements for representation, circular convolution, and dot products, we estimate that at least a total of 260000 or about 2.8 times as many neurons would be needed to obtain the same model performance without the optimization as with it.

## Discussion

We derived a mostly analytical approximation of the representational error of an NEF ensemble in dependence of its radius. This can be used to find a near optimal radius for the representation of a low-dimensional subvector of a high-dimensional semantic pointer. Doing so is an efficient operation as only the activity matrix ***A*** has to be empirically estimated. The minimum of the resulting error function can be found quickly as it is well-formed.

The method provides a number of important improvements. First, it can be used instead of the default radius which is only optimized for a specific neuron type and parameter set. Second, it allows us to obtain a radius without relying on trial and error methods or a rule of thumb. Third, fewer resources (e.g. simulated neurons) are needed to achieve the same level of performance as the error is reduced with a well-chosen radius.

Using the optimized radius yields a reduction of the RMSE up to a factor of 1.8 in the case of circular convolution and a factor of up to 25.7 in case of a dot product compared to the current Nengo default implementation. Both operations are frequently used in cognitive models built with the Semantic Pointer Architecture. On the SpiNNaker neuromorphic platform the reduction by a factor of 1.3 is more moderate, but still allowed a reduction of the number of neurons by 40%. Also, note that only 25 dimensions were used on the SpiNNaker platform. As current limitations with the hardware implementation are overcome, allowing higher dimensional semantic pointers, the usefulness of the presented method is expected to increase. In general, the variance of the individual unit vector components will decrease with increasing vector dimensionality as the distribution of vector component lengths will shift to smaller values and decrease in width. That allows for a smaller radius and increases the benefit of the radius optimization.

In cognitive models a number of these operations are often used in sequence, resulting in the accumulation of error. When a smaller error is introduced by the individual operations, it is possible to build larger cognitive networks without negative functional consequences due to accumulated error. Alternatively, a reduction in the number of neurons is possible while keeping the error constant. This allows for a more efficient use of resources, including neuromorphic hardware, to run even larger models or allow for more processing in power-sensitive applications.

More generally, we have demonstrated the potential of adapting neural network parameters to the distribution of the input signals to the specific neural subsystems. In SPA models it is common to have high-dimensional vectors split up into subvectors, consequently we focussed our optimization on this particular input structure. However, future work can focus on differently structured input which should allow for related derivations of error functions that can be optimized in a similar way.

Similarly, the analysis in this paper used the *L*_2_ norm as error measure. We expect future work to consider other cases in which different error norms might be more appropriate. The choice of norm determines the trade-off that is being made between having a majority of small errors and a few large errors versus all errors being similar in magnitude. Similar optimizations should be achievable in these cases.

## Conclusion

By considering the probability distribution of represented values within the Semantic Pointer Architecture, we were able to derive a method for determining an optimized radius for neural networks constructed using the Neural Engineering Framework. Depending on the hardware platform and calculated transformation neuron numbers could be reduced by 40% up to 97.5% while still achieving a comparable performance to unoptimized networks. Ultimately, this allows to simulate more complex networks as the hardware can be used more efficiently.

We are planning to include the proposed methods in the Nengo neural network simulator in the future.

## Supporting Information

S1 Appendix PDF of the length of a random vector with normal distributed components. (PDF)

S2 Appendix PDF of the length of a subvector of a unit vector. (PDF)

S3 Appendix Error outside of radius expressed with Beta functions. (PDF)

S4 Appendix Scaling of number of neurons with dimensions. (PDF)
