# Supplementary material for: Optimizing Semantic Pointer Representations for Symbol-Like Processing in Spiking Neural Networks
Source: PLoS One. 2016 Feb 22;11(2):e0149928. doi: 10.1371/journal.pone.0149928 (PMC4762696; doi:10.1371/journal.pone.0149928)
Supplement: S1 Appendix — (PDF) [file pone.0149928.s001.pdf]

S1 Appendix

PDF of the length of a random vector with normal distributed components.

Theorem 1. *Let* $\boldsymbol{v} = (v_1, \ldots, v_D)$ *be a vector of dimension* $D \in \mathbb{N}^+$ *with random components distributed independently according to* $v_i \sim \mathcal{N}(0;\sigma)$*. Then the probability density function of* $|\boldsymbol{v}|$ *is*

$$p_{|\boldsymbol{v}|}(x;\sigma,D) = k_D x^{D-1} \exp\left(-\frac{x^2}{2\sigma^2}\right) \tag{1}$$

with normalizing constant

$$k_D = \frac{1}{2^{(D/2)-1}\sigma^D \Gamma(\frac{D}{2})}. \tag{2}$$

Proof. The length of $\boldsymbol{v}$ is distributed according to

$$p_{|\boldsymbol{v}|}(x;\sigma,D) = \left(\frac{1}{\sqrt{2\pi\sigma^2}}\right)^D \int_{-\infty}^{\infty} \mathrm{d}v_1 \cdots \int_{-\infty}^{\infty} \mathrm{d}v_D \exp\left(-\frac{v_1^2}{2\sigma^2}\right) \cdots \exp\left(-\frac{v_D^2}{2\sigma^2}\right) \delta\left(|\boldsymbol{v}| - x\right). \tag{3}$$

By transforming to polar coordinates we obtain

$$\begin{aligned}
p_{|\boldsymbol{v}|}(x;\sigma,D) &= \left(\frac{1}{\sqrt{2\pi\sigma^2}}\right)^D \int\int_0^{\infty} \delta\left(|\boldsymbol{v}| - x\right) |\boldsymbol{v}|^{D-1} \exp\left(-\frac{|\boldsymbol{v}|^2}{2\sigma^2}\right) \mathrm{d}|\boldsymbol{v}|\, \mathrm{d}\Omega_D \\
&= \left(\frac{1}{\sqrt{2\pi\sigma^2}}\right)^D \int x^{D-1} \exp\left(-\frac{x^2}{2\sigma^2}\right) \mathrm{d}\Omega_D \\
&= \left(\frac{1}{\sqrt{2\pi\sigma^2}}\right)^D \cdot \frac{2\pi^{D/2}}{\Gamma\left(\frac{D}{2}\right)} \cdot x^{D-1} \exp\left(-\frac{x^2}{2\sigma^2}\right) \\
&= \frac{1}{2^{(D/2)-1}\sigma^D \Gamma\left(\frac{D}{2}\right)} \cdot x^{D-1} \exp\left(-\frac{x^2}{2\sigma^2}\right)
\end{aligned} \tag{4}$$

with the D-dimensional solid angle differential $\mathrm{d}\Omega_D$. ☐
