# Supplementary material for: Optimizing Semantic Pointer Representations for Symbol-Like Processing in Spiking Neural Networks
Source: PLoS One. 2016 Feb 22;11(2):e0149928. doi: 10.1371/journal.pone.0149928 (PMC4762696; doi:10.1371/journal.pone.0149928)
Supplement: S2 Appendix — (PDF) [file pone.0149928.s002.pdf]

S2 Appendix

PDF of the length of a subvector of a unit vector.

Lemma 1. *Given a random variable X with probability distribution function $p_X(x)$, the probability distribution function of $Y = X^2$ is given by*

$$p_Y(y) = \frac{1}{2\sqrt{y}} \cdot p_X(\sqrt{y}). \tag{1}$$

Proof. Performing a change in variable with $y = f(x) = x^2$ gives

$$\begin{aligned} p_Y(y) &= \left|\tfrac{\mathrm{d}}{\mathrm{d}y}\left(f^{-1}(y)\right)\right| \cdot p_X(f^{-1}(y)) \\ &= \left|\tfrac{\mathrm{d}}{\mathrm{d}y}\left(\sqrt{y}\right)\right| \cdot p_X(\sqrt{y}) \\ &= \frac{1}{2\sqrt{y}} \cdot p_X(\sqrt{y}). \end{aligned} \tag{2}$$

□

Theorem 1. *Let $\boldsymbol{v} = (v_1, \ldots, v_{n+m})$ be a vector of dimension $n+m$ with $n, m \in \mathbb{N}^+$ and random components distributed independently according to $v_i \sim \mathcal{N}(0; \sigma)$. Let $\hat{\boldsymbol{v}} = \boldsymbol{v}/|\boldsymbol{v}|$ be the vector normalized to unit length. Then the length $|\hat{\boldsymbol{v}}_{1:m}|$ of a subvector $\hat{\boldsymbol{v}}_{1:m} = (\hat{v}_1, \ldots, \hat{v}_m)$ with m components is distributed according to the probability distribution function*

$$p_{\mathrm{SB}}(x; n, m) = \frac{2}{\mathrm{B}\left(\frac{n}{2}, \frac{m}{2}\right)} \left(x^2\right)^{(m-1)/2} \left(1 - x^2\right)^{n/2-1}. \tag{3}$$

Proof. The length of the subvector is given by the random variable

$$\begin{aligned} |\hat{\boldsymbol{v}}_{1:m}| &= \frac{|\boldsymbol{v}_{1:m}|}{|\boldsymbol{v}|} \\ &= \frac{|\boldsymbol{v}_{1:m}|}{\sqrt{|\boldsymbol{v}_{1:m}|^2 + |\boldsymbol{v}_{m+1:n+m}|^2}} \\ &= \frac{1}{\sqrt{1 + \frac{|\boldsymbol{v}_{m+1:n+m}|^2}{|\boldsymbol{v}_{1:m}|^2}}} \\ &= \frac{1}{\sqrt{1+Q}} \end{aligned} \tag{4}$$

with random variable

$$Q = \frac{|\boldsymbol{v}_{m+1:n+m}|^2}{|\boldsymbol{v}_{1:m}|^2}. \tag{5}$$

The probability distribution function of the quotient of two random variables is given by

$$p_{X/Y}(x) = \int_{-\infty}^{\infty} |y|\, p_X(xy) p_Y(y)\, \mathrm{d}y \tag{6}$$

Using this equation, Theorem 1 in S1 Appendix, and Lemma 1 we obtain

$$\begin{aligned} p_Q(x;\sigma,n,m) &= \int_{-\infty}^{\infty} |y|\, p_{|\boldsymbol{v}_{m+1:n+m}|^2}(xy;\sigma,n) p_{|\boldsymbol{v}_{1:m}|^2}(y;\sigma,m)\,\mathrm{d}y \\ &= \int_{0}^{\infty} \frac{|y|}{4\sqrt{xy^2}} \cdot p_{|\boldsymbol{v}_{m+1:n+m}|^2}(\sqrt{xy};\sigma,n) p_{|\boldsymbol{v}_{1:m}|}(\sqrt{y};\sigma,m)\,\mathrm{d}y \\ &= \int_{0}^{\infty} \frac{|y|}{4\sqrt{xy^2}} \cdot k_n k_m (\sqrt{xy})^{n-1} (\sqrt{y})^{m-1} \exp\left(-\frac{xy}{2\sigma^2}\right) \exp\left(-\frac{y}{2\sigma^2}\right)\mathrm{d}y \\ &= \frac{k_n k_m}{4\sqrt{x}} \int_{0}^{\infty} x^{(n-1)/2} y^{(n+m-2)/2} \exp\left(-\frac{x+1}{2\sigma^2}\cdot y\right)\mathrm{d}y \\ &= \frac{k_n k_m}{4} \cdot x^{n/2-1} \cdot \frac{2\sigma^2}{x+1} \int_{0}^{\infty} \underbrace{\frac{z+1}{2\sigma^2}}_{\phi'(y)} \underbrace{y^{(n+m-2)/2} \exp\left(-\frac{x+1}{2\sigma^2}\cdot y\right)}_{f(\phi(y))}\mathrm{d}y. \end{aligned} \tag{7}$$

Applying a substitution with $\phi(y) = \frac{x+1}{2\sigma^2} \cdot y$ and $f(\phi(y)) = \left(\frac{2\sigma^2}{x+1} \cdot \phi(y)\right)^{(n+m-2)/2} \cdot \exp\bigl(-\phi(y)\bigr)$ gives

$$\begin{aligned} p_Q(x;\sigma,n,m) &= \frac{k_n k_m}{4} \cdot x^{n/2-1} \cdot \frac{2\sigma^2}{x+1} \cdot \\ &\quad \int_{\phi(0)}^{\phi(\infty)} \left(\frac{2\sigma^2}{x+1}\cdot\phi(y)\right)^{(n+m-2)/2} \cdot \exp\bigl(-\phi(y)\bigr)\,\mathrm{d}y \\ &= \frac{k_n k_m}{4} \cdot x^{n/2-1} \cdot \left(\frac{2\sigma^2}{x+1}\right)^{(n+m)/2} \int_{0}^{\infty} \phi(y)^{(n+m-2)/2} \cdot \exp\bigl(-\phi(y)\bigr)\,\mathrm{d}y \\ &= \frac{k_n k_m}{4} \cdot x^{n/2-1} \cdot \left(\frac{2\sigma^2}{x+1}\right)^{(n+m)/2} \Gamma\left(\frac{n+m}{2}\right). \end{aligned} \tag{8}$$

Another change in variables is performed to obtain $p_{\mathrm{SB}}(x; n, m)$ with $x = f(y) = \sqrt{\frac{1}{1+y}}$.

$$
\begin{aligned}
p_{\mathrm{SB}}(x; n, m) &= \left| \frac{\mathrm{d}}{\mathrm{d}x} \left(f^{-1}(x) \right) \right| \cdot p_Q \left(f^{-1}(x); \sigma, n, m \right) \\
&= \left| -2x^{-3} \right| \cdot \frac{k_n k_m}{4} \cdot \left(x^{-2} - 1 \right)^{n/2-1} \cdot \left(\frac{2\sigma^2}{x^{-2}} \right)^{(n+m)/2} \Gamma\left(\frac{n+m}{2} \right) \\
&= \frac{k_n k_m}{2} \cdot \left(2\sigma^2 \right)^{(n+m)/2} \cdot x^{n+m-3} \left(x^{-2} - 1 \right)^{n/2-1} \Gamma\left(\frac{n+m}{2} \right) \\
&= \frac{\left(2\sigma^2 \right)^{(n+m)/2} \cdot \Gamma\left(\frac{n+m}{2} \right)}{2 \cdot 2^{(n/2)-1} \cdot 2^{(m/2)-1} \cdot \sigma^n \sigma^m \cdot \Gamma\left(\frac{n}{2} \right) \Gamma\left(\frac{m}{2} \right)} \cdot x^{n+m-3} \left(x^{-2} - 1 \right)^{n/2-1} \\
&= \frac{2}{\mathrm{B}\left(\frac{n}{2}, \frac{m}{2} \right)} \cdot x^{n+m-3} \left(x^{-2} - 1 \right)^{n/2-1} \\
&= \frac{2}{\mathrm{B}\left(\frac{n}{2}, \frac{m}{2} \right)} \cdot x^{m-1} \left(1 - x^2 \right)^{n/2-1} \\
&= \frac{2}{\mathrm{B}\left(\frac{n}{2}, \frac{m}{2} \right)} \cdot \left(x^2 \right)^{(m-1)/2} \left(1 - x^2 \right)^{n/2-1}
\end{aligned}
\tag{9}
$$

□

Corollary 1. *The squared length* $|\hat{\boldsymbol{v}}_{1:m}|^2$ *of the subvector is distributed according to a Beta distribution with* $\alpha = m/2,\ \beta = n/2$.

Theorem 2. *If* X *is a random variable distributed according to* $X \sim p_{\mathrm{SB}}(x; n, m)$, *then the cumulative distribution function is*

$$
F_{\mathrm{SB}}(x; n, m) = \frac{\mathrm{B}\left(x^2; \frac{m}{2}, \frac{n}{2} \right)}{\mathrm{B}\left(\frac{m}{2}, \frac{n}{2} \right)}. \tag{10}
$$

Proof.

$$
\begin{aligned}
F_{\mathrm{SB}}(x; n, m) &= \int_0^x \frac{2}{\mathrm{B}\left(\frac{n}{2}, \frac{m}{2} \right)} \cdot \left(x^2 \right)^{(m-1)/2} \left(1 - x^2 \right)^{n/2-1} \mathrm{d}x \\
&= \frac{1}{\mathrm{B}\left(\frac{n}{2}, \frac{m}{2} \right)} \cdot \int_0^x 2x \cdot x^{-1} \cdot \left(x^2 \right)^{(m-1)/2} \left(1 - x^2 \right)^{n/2-1} \mathrm{d}x \\
&= \frac{1}{\mathrm{B}\left(\frac{n}{2}, \frac{m}{2} \right)} \cdot \int_0^x \underbrace{2x}_{\phi'(x)} \cdot \underbrace{\left(x^2 \right)^{m/2-1} \left(1 - x^2 \right)^{n/2-1}}_{f(\phi(x))} \mathrm{d}x
\end{aligned}
\tag{11}
$$

Applying a substitution with $\phi(x) = x^2$ gives:

$$
\begin{aligned}
F_{\mathrm{SB}}(x; n, m) &= \frac{1}{\mathrm{B}\left(\frac{n}{2}, \frac{m}{2} \right)} \cdot \int_{\phi(0)}^{\phi(x)} \left(\phi(x) \right)^{m/2-1} \left(1 - \phi(x) \right)^{n/2-1} \mathrm{d}\phi(x) \\
&= \frac{\mathrm{B}\left(x^2; \frac{m}{2}, \frac{n}{2} \right)}{\mathrm{B}\left(\frac{m}{2}, \frac{n}{2} \right)}
\end{aligned}
\tag{12}
$$

□
