# Supplementary material for: Optimizing Semantic Pointer Representations for Symbol-Like Processing in Spiking Neural Networks
Source: PLoS One. 2016 Feb 22;11(2):e0149928. doi: 10.1371/journal.pone.0149928 (PMC4762696; doi:10.1371/journal.pone.0149928)
Supplement: S3 Appendix — (PDF) [file pone.0149928.s003.pdf]

S3 Appendix

Error outside of radius expressed with Beta functions.
The error from points falling outside the radius is given by

$$E_{x>r} = \frac{\int_r^1 (y-r)^2 \, p_{\mathrm{SB}}(y; D-m, m)\,\mathrm{d}y}{1 - F_{\mathrm{SB}}(x; D-m, m)}. \tag{1}$$

The numerator of that equation can be split up as

$$\begin{aligned} \int_r^1 (y-r)^2 \, p_{\mathrm{SB}}(y; D-m, m)\,\mathrm{d}y = & \int_r^1 y^2 \cdot p_{\mathrm{SB}}(y; D-m, m)\,\mathrm{d}y \\ & - 2r \int_r^1 y \cdot p_{\mathrm{SB}}(y; D-m, m)\,\mathrm{d}y \\ & + r^2 \int_r^1 p_{\mathrm{SB}}(y; D-m, m)\,\mathrm{d}y \end{aligned} \tag{2}$$

to calculate the individual integrals as

$$\begin{aligned} \int_r^1 y^2 \cdot p_{\mathrm{SB}}(y; D-m, m)\,\mathrm{d}y &= \frac{2}{\mathrm{B}\left(\frac{D-m}{2}, \frac{m}{2}\right)} \cdot \int_r^1 \left(y^2\right)^{(m-1)/2} \left(1-y^2\right)^{(D-m-2)/2} \mathrm{d}y \\ &= \frac{1}{\mathrm{B}\left(\frac{D-m}{2}, \frac{m}{2}\right)} \cdot \int_{r^2}^1 \left(\phi(y)\right)^{m/2} \left(1-\phi(y)\right)^{(D-m-2)/2} \mathrm{d}\phi(y) \\ &= \frac{1}{\mathrm{B}\left(\frac{D-m}{2}, \frac{m}{2}\right)} \left[\int_0^1 \left(\phi(y)\right)^{m/2} \left(1-\phi(y)\right)^{(D-m-2)/2} \mathrm{d}\phi(y) \right. \\ & \qquad \left. - \int_0^{r^2} \left(\phi(y)\right)^{m/2} \left(1-\phi(y)\right)^{(D-m-2)/2} \mathrm{d}\phi(y) \right] \\ &= \frac{\mathrm{B}\left(\frac{m}{2}+1, \frac{D-m}{2}\right) - \mathrm{B}\left(r^2; \frac{m}{2}+1, \frac{D-m}{2}\right)}{\mathrm{B}\left(\frac{D-m}{2}, \frac{m}{2}\right)} \end{aligned} \tag{3}$$

$$
\begin{aligned}
\int_r^1 y \cdot p_{\mathrm{SB}}(y; D-m, m)\,\mathrm{d}y &= \frac{2}{\mathrm{B}\left(\frac{D-m}{2}, \frac{m}{2}\right)} \cdot \int_r^1 y \left(y^2\right)^{(m-1)/2} \left(1-y^2\right)^{(D-m-2)/2} \mathrm{d}y \\
&= \frac{1}{\mathrm{B}\left(\frac{D-m}{2}, \frac{m}{2}\right)} \cdot \int_{r^2}^1 \left(\phi(y)\right)^{(m-1)/2} \left(1-\phi(y)\right)^{(D-m-2)/2} \mathrm{d}\phi(y) \\
&= \frac{1}{\mathrm{B}\left(\frac{D-m}{2}, \frac{m}{2}\right)} \left[\int_0^1 \left(\phi(y)\right)^{(m-1)/2} \left(1-\phi(y)\right)^{(D-m-2)/2} \mathrm{d}\phi(y) \right. \\
&\quad \left. - \int_0^{r^2} \left(\phi(y)\right)^{(m-1)/2} \left(1-\phi(y)\right)^{(D-m-2)/2} \mathrm{d}\phi(y)\right] \\
&= \frac{\mathrm{B}\left(\frac{m+1}{2}, \frac{D-m}{2}\right) - \mathrm{B}\left(r^2; \frac{m+1}{2}, \frac{D-m}{2}\right)}{\mathrm{B}\left(\frac{D-m}{2}, \frac{m}{2}\right)}
\end{aligned} \tag{4}
$$

$$
\begin{aligned}
\int_r^1 p_{\mathrm{SB}}(y; D-m, m)\,\mathrm{d}y &= 1 - F_{\mathrm{SB}}(r; D-m, m) \\
&= \frac{\mathrm{B}\left(r^2; \frac{m}{2}, \frac{D-m}{2}\right)}{\mathrm{B}\left(\frac{m}{2}, \frac{D-m}{2}\right)}.
\end{aligned} \tag{5}
$$

Thus, the integral can be expressed purely with beta functions.
