# Supplementary material for: Optimizing Semantic Pointer Representations for Symbol-Like Processing in Spiking Neural Networks
Source: PLoS One. 2016 Feb 22;11(2):e0149928. doi: 10.1371/journal.pone.0149928 (PMC4762696; doi:10.1371/journal.pone.0149928)
Supplement: S4 Appendix — (PDF) [file pone.0149928.s004.pdf]

S4 Appendix

Scaling of number of neurons with dimensions. The number of neurons in a single NEF ensemble has to be scaled with D^2 where D is the number of dimensions to keep the absolute error from noise constant. In contrast, if each vector component is represented in a single ensemble the number of neurons has only be scaled linearly with D if the ensemble radii are set proportional to $1/\sqrt{D}$ which corresponds to the Nengo default scaling of $3.5/\sqrt{D}$. To show this, we ran simulations with both approaches for different dimensionalities with a total of $20D$ and $20D^2$ neurons. The input value to the ensembles was a null vector and mean absolute squared distance of the decoded vector was recorded for one second of simulated time. For each choice of D a total of 20 trials was performed. The results are shown in Fig 1 and confirm the initial statement.

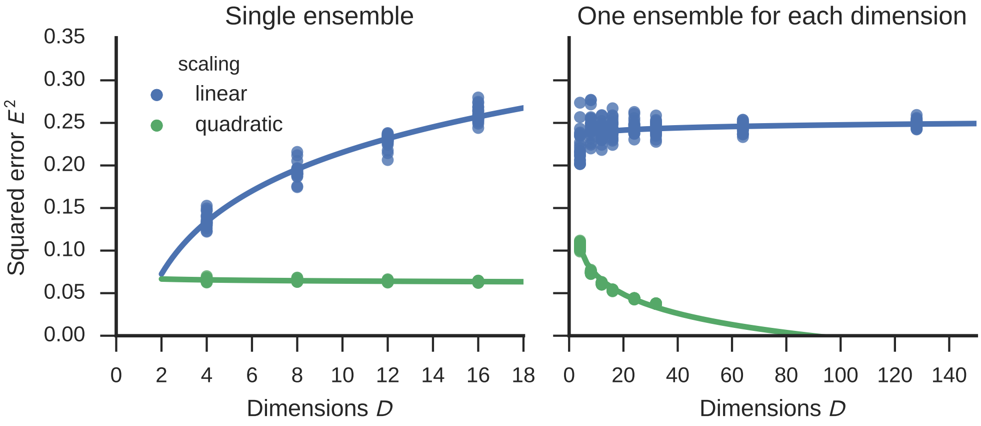

Figure 1: **Squared error in dependence of vector dimensionality and scaling of neuron number.** In the left panel the vector was represented in a single ensemble; in the right panel each vector component was represented in a separate ensemble with the radius set to the Nengo default of $3.5/\sqrt{D}$. Each point is the mean error in one trial run. The curves are linear regressions of the form $E^2 \sim \log(D)$. Note that the two plots use a different x-scale as single ensemble simulations are costly for a large number of dimensions.
